# A New Secure Model for Data Protection over Cloud Computing

**DOI:** 10.1155/2021/8113253

**Published:** 2021-11-24

**Authors:** Amr M. Sauber, Passent M. El-Kafrawy, Amr F. Shawish, Mohamed A. Amin, Ismail M. Hagag

**Affiliations:** ^1^Faculty of Science, Menoufia University, Shibin El Kom, Egypt; ^2^School of Information Technology and Computer Science, Nile University, Sheikh Zayed, Egypt; ^3^EL Madina Higher Instute of Admininstration and Technology, Giza, Egypt

## Abstract

The main goal of any data storage model on the cloud is accessing data in an easy way without risking its security. A security consideration is a major aspect in any cloud data storage model to provide safety and efficiency. In this paper, we propose a secure data protection model over the cloud. The proposed model presents a solution to some security issues of cloud such as data protection from any violations and protection from a fake authorized identity user, which adversely affects the security of the cloud. This paper includes multiple issues and challenges with cloud computing that impairs security and privacy of data. It presents the threats and attacks that affect data residing in the cloud. Our proposed model provides the benefits and effectiveness of security in cloud computing such as enhancement of the encryption of data in the cloud. It provides security and scalability of data sharing for users on the cloud computing. Our model achieves the security functions over cloud computing such as identification and authentication, authorization, and encryption. Also, this model protects the system from any fake data owner who enters malicious information that may destroy the main goal of cloud services. We develop the one-time password (OTP) as a logging technique and uploading technique to protect users and data owners from any fake unauthorized access to the cloud. We implement our model using a simulation of the model called Next Generation Secure Cloud Server (NG-Cloud). These results increase the security protection techniques for end user and data owner from fake user and fake data owner in the cloud.

## 1. Introduction

Cloud computing has changed the way of delivering computing services [1]. In outsourcing computation models, an increasing number of susceptible devices (customers) rely upon faraway servers (nodes) for information storage and computations [2]. Increasing number of cloud data centers all over the world is consuming a vast amount of power [3]. Cloud computing is evolving as a brand new fashion of complete disbursed computing. It has moved the computation away from domestic PCs and small companies to large-scale information facilities and made it fantastic for clients and IT companies with the aid of using blocking big quantity of capital investments. A great deal of cloud computing research has been concerned over some issues and challenges that based on behind the rule of cloud computing [4]. The cloud computing (CC) is a provider that is added through information centers, which are primarily based totally on virtualization technologies. Cloud computing facilitates collaboration, communication, and essential online services during the COVID-19 crisis [5].

Scientific collaborations executing such experiments have numerous wishes and undertake distinctive methods in growing the computing frameworks [6]. Big data may exist in a huge number of small files, so significant function of the cloud data warehouse is to make sure of the security of confidential information that may be performed through strategies of steganography and cryptography [7]. There are problems of security including information loss, integrity, and botnet posing extreme threats to companies' information and software [8]. Security of sensitive data is a pressing need in trendy communication, mainly in cloud [9, 10]. Every day, the number of people using cloud computing services increases, and lots of data have been stored in cloud computing environments. Cloud computing has giant blessings that consist of remote storage, mobility, information sharing, value financial savings in hardware and software, etc. [11].

Data leakage to cloud services is also increasing every year because of attackers who are always trying to exploit the security vulnerabilities of cloud. Engineers and researchers try to identify the possible cloud threats and attacks in order to implement better security mechanisms to protect sensitive data and cloud computing environments [12]. Recently, many data secure models over the cloud computing were proposed [13–17].

Moving applications to the cloud and gaining access to the blessings is a way of first comparing unique records safety problems and cloud security problems. When businesses circulate programs from on-premise to cloud-based ones, demanding situations rise up from records residency, enterprise compliance requirements, and privacy and third-party celebration duties regarding the remedy of sensitive information. Corporate regulations or the policies of the governing jurisdictions affect the manner sensitive information is controlled, consisting of where it is far located, what kinds of data may be gathered and saved, and who has the right of entry to it. These problems can decide the diploma to which companies can recognize the price of cloud computing.

A new superior framework of security for client identity, which incorporates aspect authentication, AES primarily based totally document encryption and decryption of information uploaded over cloud computing, admin verification and locking of users, fetching IP info of clients, and disbursed database garage, i.e., statistics, is saved in ranges; this means that consumer login info is saved in a single database, and encryption/decryption of info such as file uploaded and key is saved on numerous databases. One aspect authentication is at risk of password guessing due to the fact that humans do not regularly extrade their password. Therefore, security of the cloud computing is critical in the modern-day world. Overall, paintings offer improving security for cloud computing, as well as protection and security for complete cloud-based computing structure [18].

Open information sharing with others is feasible with cloud. Moving data to a third-party (cloud carrier provider) off-web website online garage community wherein information proprietors have little manage affords distinct problems to privacy–threats of illegal disclosure of sensitive information via carrier providers, facts integrity authenticity of out-of-carrier information, etc. The cloud allows the alternate of facts; cautious interest ought to be paid to the complete get admission to manage of the saved information. This touchy fact about confidentiality is an everyday method to encrypt it till its miles moved to the cloud computing. The consumer encrypts his document and shops it in a conventional public key infrastructure at the cloud server, and the simplest real permitted consumer is instructed approximately about the decryption key. With regard to confidentiality, this method is secure; however, reliable, examined, and complicated control and distribution are essential for this solution. Even this solution would not succeed, because the variety of software customers is growing.

Our contributions are as follows:

1. The technique of our proposed scheme proves the identity of the users authorized without the need to reveal their passwords. We also developed OTP logging and registration techniques to overcome fake identity issue. We increase the encryption data of users and data owners by merging secret key and private key when the user accesses the data in the cloud.
2. We find new solution for threats and attacks over cloud computing to provide the security and safety of the cloud and its users. We discuss a number of possible security measures, which should be considered in any cloud-based model. Also, we cover some security functions, which should be in a model as a guarantee for safety checks of a system and its users and the protection of private and sensitive data for all users of the cloud.
3. Our model can solve many attacks such as replay attack, insider attacks, and MITM. Moreover, the work enjoys many features as follows: (1) it supports mutual authentication between authentication cloud server and a user; (2) it offers user anonymity; (3) in our scheme, the password is saved in the service provider. Thus, the conﬁrmation cost of the security-sensitive table is decreased.
4. The proposed model overcomes the balancing between security and usability according to client demand.
5. We implement the proposed model by NG-Cloud simulation to overcome the security risks defined by the security functions over cloud computing such as authentication, authorization, and privacy.

The rest of our paper is organized as follows.  Section 2 explains related work.  Section 3 describes our proposed model of data sharing. Implementation and experimental results and analysis of the proposed model are described in Sections 4 and 5. Finally, the conclusion is described in Section 6.

## 2. Related Work

In 2014, Xin Dong et al. [13] (Dong's scheme) proposed a data sharing, fueled by favorable trends in cloud. A cipher-text policy attribute based encryption (CP-ABE) [19] combined with identity-based encryption (IBE) technique [20] is utilized in Dong's scheme. Supported and secure dynamic operations, such as file creation, user revocation, and modification, were included in Dong's scheme. Dong's model consists of four parties in the network:

1. The data owner, who has data stored in the cloud and depends on the cloud for data maintenance
2. Data owner can be enterprises or individual customers
3. The data user, who accesses the data shared by the data owner, downloads data of interest, and decrypts it using his secret keys
4. The private key generator (PKG), which delivers public keys to the data owner and generates and distributes corresponding private keys to the users

Xin Dong's scheme is specialized in supplying a reliable and stable cloud information sharing carrier that permits customers to dynamically get the right of entry to their information. Privacy-maintaining information coverage with semantic protection is proposed inside the scheme, with the aid of using cipher-text policy attribute-primarily based encryption (CP-ABE) mixed with identity-based encryption (IBE) techniques.

Although Dong's scheme enabled only the authorized users to access and restore files correctly, the scheme did not solve the problem with the fake identity; i.e., when an authorized user reveals user name and password to an unauthorized user, the system cannot know if he/she is an authorized client or not. This problem is an important requirement for security over cloud, because in case of unauthorized client access to the system security violation cannot be accepted. Moreover, this system suffers from allowing fake data owner, who uploads malicious data or files on the cloud server. Fake data owner can enter malicious data on the cloud and upload harmful viruses that can destroy the data stored on the cloud server.

OTP is described as random code generated through cloud server, and then, this code is sent to the clients or data owners through e-mail address and finally sent to the mobile in the phases of logging and uploading, respectively. In our proposed model, we solve the previous problems through using technique one-time password (OTP) in two phases. The two phases are the process of user login and the process of uploading files on the cloud computing environment.

Getaneh Berie Tarekegn et al. gave a better understanding of the cloud, to evaluate how privacy and security problems occur in the context of cloud computing and discuss the mechanisms to address these problems in cloud computing. Therefore, security has always been the basic problem for IT executives when it comes to cloud adoption. Cloud computing is an agglomeration of methods, operating systems, storage, networking, and virtualization, each fraught with inherent security problems. Cloud computing is a novel technique with shared resources and lower cost and is based on “pay per utilize” according to the customer demand. Due to more characteristics, it has an effect on IT budget and also affects security and privacy.

A privacy steerage committee ought to additionally be created to assist make selections associated with information privacy. This committee will make sure that your business enterprise is ready to satisfy the statistics privacy needs of its customers and regulators. Information with inside the cloud is normally globally disbursed, which increases worries approximately jurisdiction, statistics publicity, and privacy. Organizations stand a chance of no longer complying with authorities' guidelines as might be defined similarly at the same time as the cloud carriers who disclose touchy information chance with legal ability. The essential assignment for software program engineers is to lay out cloud offerings in this sort of manner as to lower privacy chance and to ensure felony compliance. Security and privacy issues gift a sturdy barrier for customers to evolve into cloud computing systems. This study offers an outline concerning characteristics, features, security-architecture, and threats, assaults, and current solutions [21].

Eesa Alsolami stated multiple problems with cloud computing that impair security and privacy of information and presented a threat that impacts data residing in the cloud. There are different mitigating models for countering these threats that are introduced here, as well as multiple open problems that are noted in this research for more researches for providing a secure cloud computing environment [22].

That research explained security threats and privacy problems for data in cloud computing environment; some examples of these problems are as follows:

(1)  Privileged user access: cloud service providers usually have limitless access to the data on user for privileged users, which is a potentially high-risk factor that can lead to unethical access to user data.
(2) Privileged user access: in this issue, cloud service providers generally have countless access to the data on user for privileged clients that are a probably high-hazard component that could result in unethical entry to client information.
(3) Protective monitoring: the customers have constrained ability to perform their very own defensive techniques because of complicated shape of cloud and needed to base on cloud provider company for defensive monitoring, which is like an invasion to privacy.
(4) Data leakage and inconsistency: it commonly happens in cloud, while users information is time and again saved at more than one information facility for backup. This allotted storage would increase the chance of information leakage, and synchronization screw-ups cause information inconsistency.
(5) Nonsecure interoperation: multidomain gets the right of entry to manage cloud platform additionally and poses security attacks to customer data saved in cloud because of interoperation on shared resources, which requires international guidelines for mediating all customers getting right of entry and pleasing all customer requirements.
(6) Encryption algorithms: presently, encryption is a basic solution in explaining information privacy problems with inside the cloud computing environment. With encryption algorithms, exclusive data is encrypted and is handiest, accessed through clients having encryption keys.
(7) Trust management: trust control is one promising method for addressing safety and privacy problems with inside the cloud computing environment, its miles specially classified as smooth and tough agree with control. Soft agree with control is associated with defining dating among events appearing in any action. Hard agree with control is an upcoming fashion for resolving privacy issues and records integrity issues, because it is offered with digital infrastructure furnished to the user, which is commonly constructed on nonsteady bodily hardware.

It is proposed that duty of cloud carrier vendors has to be elevated in subjects associated with safety and privacy problems, in order for suitable countermeasures to be deployed for customer information protection. One of the critical challenging situations for safety in cloud is compromised software program interfaces, which might be applied to engage with cloud offerings, exposing more than one security problem that needs extra evaluation over integrating safety offerings in the course of the platform. Another problem for cloud computing is information segregation, which requires extra researches to be performed at the hassle of steady data storage. In addition to this, hassle like steady consumer access, hacking, data leakage, and absence of short recuperation protocols needs extra research to be performed to absolutely examine those issues [22].

In Srijita Basu et al.'s study, protection in cloud is taken into consideration; it must now no longer be confined inside the limits of information protection; however, the corresponding virtual machine (VM) protection must additionally be taken into consideration equally. Hence, the primary consciousness of that paper is to categorize the cloud protection into appropriate domain names and discover the cautioned solutions. This paper provides the primary protection loopholes, in addition to protection necessities of a present cloud system. A generalized view of those issues was given to enhance the significance of expertise regarding the security flaws of the cloud computing version and devising appropriate countermeasures for them. Therefore, diverse cloud protection schemes were defined on a comparative model. Overall, the studies aim at building a right photograph of the prevailing state of scenarios and extra potentialities of cloud protection [23].

In Feras Awaysheh et al.'s study, big data security plays a decisive function in the enormous adoption of cloud architectures. However, it is challenging to advance a complete safety diagram until it is based totally on a preliminary evaluation that ensures a sensible impenetrable assembly and addresses domain-specific vulnerabilities. It afforded a novel security-by-design framework for BD frameworks deployment over cloud. In particular, it depends on a systematic protection evaluation methodology and a completely automated security evaluation framework. It addressed the big cloud-specific protection demands to absolutely illustrate the number protection elements in a conceptual context. This framework permits the mapping of big cloud security domain information to the exceptional practices in the plan Step [24].

## 3. Proposed Model

### 3.1. The Methodology of the Proposed Model

One of the serious issues in cloud computing is fake data. Fake data can propose fewer issues perturbing actual objects. For instance, the agents are hospital, and medical records are distributed data objects. In this case, even small modifications to the data of real patients may be undesirable. Hence, the addition of a fake clinical data may be acceptable, due to the fact that no affected person fits those data, and consequently no patient would ever be dealt with primarily based on fake data. In this case study, organization A sells to organization B a mailing list to be utilized once. Organization A provides hint data that consist of addresses owned via way of means of organization A. Thus, on every occasion, organization B utilizes the purchased mailing list; A gets copies of the mailing. These data are types of fake objects that assist in perceiving wrong use of data. Cloud computing lets in computing devices, with much less regard to their capacities and sizes, to maintain getting access to distinct types of offerings over the Internet.

Dealing with information encryption, key control is the maximum complex issue of any protection framework and network. Key control is the method of safeguarding encryption keys from loss, unauthorized access, and corruption. However, key control is normally the most cause encryption that is not always being finished via organizations. The crucial issue is the password or the safety key. If the assigned password is misplaced at some stage in the method of encryption with inside the cloud computing, there is no manner to salvage the statistics. Another trouble is approximately passwords where clients create non-unusual place words, which include their electronic mail passwords or spouse's name. The less complicated the safety key to guess, the less complicated the statistics to be breached.

**Figure 1:**
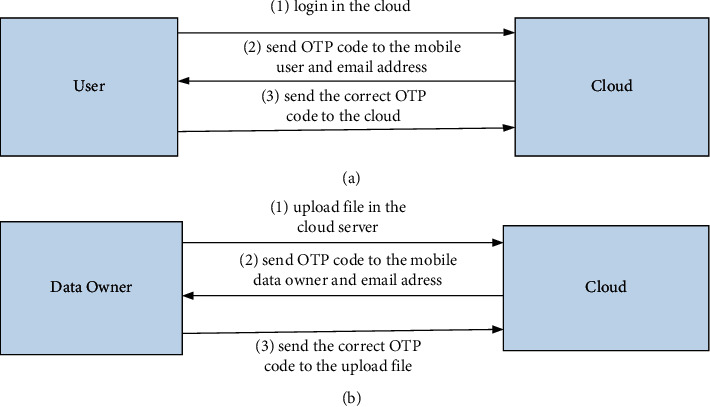
(a) The process of logging user on the cloud. (b) The process of uploading file on the cloud OTP.

The user set (US), a cloud server (CS), and data owner (DO) are basic components of our proposed model. OTP is a logging method in order to protect the users from a fake authorized access to the cloud computing environment. In case any user registers to the cloud computing environment, then the cloud will request to send an OTP code text to his private e-mail and/or his private phone number. The received OTP code correctly to the cloud to verify his identity as an authorized user should be entered by each user. We added the OTP login between the users and the cloud computing to prevent any unauthorized user to access our system illegally in our model. OTP in the proposed model makes the login process securely to protect users and their private data from any violations.  Figure 1(a) illustrates the OTP process, while a user downloads files from the cloud.

Nevertheless, a data owner may upload encrypted files on the cloud. We should protect data owners from any fake data. The fake data owner can enter malicious information that may destroy the main goal of cloud services. When data owner wants to upload files on the cloud, the cloud requests OTP code text before files upload. OTP code shall be sent to every data owner through his private e-mail and/or SMS to his private phone number. The process of uploading file to the cloud should protect encrypted data from any attacks or threats. OTP process protects data owner while uploading data to the cloud.  Figure 1(b) illustrates the OTP process while uploading files from the data owner to the cloud.

### 3.2. The Phases of Proposed Model

PKG in our model is trusted third party (TTP) structure, which facilitates interactions between users and data owners, who both trust the third party. Data owners can be a person or organization. PKG checks all critical procedure communications between user and data owner. In this model, the relying parties (user and data owner) use this trust to secure their own interactions.

**Figure 2:**
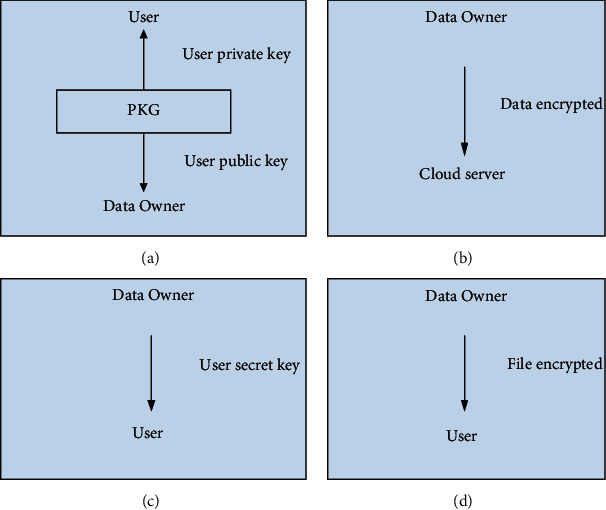
Phases for our model. (a) Phase 1. (b) Phase 2. (c) Phase 3. (d) Phase 4.

Figure 2 illustrates all phases in the proposed model stating from the process of registration and logging down to the process of obtaining the services. The proposed model consists of four phases, which achieves the security considerations mentioned above. The four phases are listed as follows.

#### 3.2.1. Phase 1: Data Owner vs User

PKG generates each user public key and their corresponding private key. Each user can download a public/private key pair, where the public key is a user ID. PKG gives the data owner a user public key (User ID) and his corresponding private key. The user ID may be a user identity card number, e-mail address, etc. Attackers can easily obtain the user ID (public key) somewhere. So, the communication channels between users and cloud server (CS) should be secured. Users can register for a cloud account through the data owner. This is to trust the relationship between the user and the data owner more significantly. The user can decrypt the encrypted data through their private key.

#### 3.2.2. Phase 2: Data Owner vs Cloud Users

Uploading data on the server is done with enough description of what type of data it contains in order to help users to know file content. The process of uploading data to the cloud is a very important phase in the safety of the entire system. Data owners encrypt the data files and assign different access privileges for each user to their data. Data owner provides secret key (SK) for encrypting file. Data owner selects user public key and provides file description. When sending the file to the user, it is encrypted with SK. We will encrypt the data by SK and public key (PK). Data owner encrypts data using symmetric key (AES), where the same key is used to encrypt and decrypt the encrypted data. Encrypted data on the cloud provide the security over cloud computing for users. When the data owner wants to upload data on the cloud server, he should encrypt data before uploading on the cloud for providing the security of data from any attackers.

#### 3.2.3. Phase 3: Cloud vs Data Owner

Cloud should ensure the data owner before uploading files, by using the OTP process. Also, cloud should support dynamic requests of data owner (e.g., adding or revoking access privileges to users and allowing them to create or delete their data). When impersonating the identity of data owner and accessing the cloud, it can upload the files. In this case, it can download malicious files, or viruses destroy the safety of cloud computing. Therefore, it emphasizes the identity of data owner when it uploads data necessary to ensure that the absence of any fake data owner is not allowed to access the cloud server. OTP will solve this problem as will be explained in detail later regarding the owner of the data.

#### 3.2.4. Phase 4: Cloud vs User

Cloud should ensure the users before downloading files, by using the OPT process. The data owner stores the encrypted files on the cloud by the secret key. The data owner gives each user a secret key via cloud. When a user wants to decrypt a file, he must obtain the file's secret key. The secret key is not enough to decrypt the files; however, users can decrypt by the secret key and the privet key paired together. The user will obtain the encrypted files from the cloud server (CS). Then, each user can decrypt the files by the file's secret key and his private key. This stage is an important stage, because, here, the user achieves the service offered from cloud computing. If an authorized user decrypts data by only secret key, probably an unauthorized user or attacker can decrypt the data if he knew the secret key. So, we find the process of decrypting data through each user difficult, and we can decrypt data by secret key and private key together to protect the data from any attacks or unauthorized user. This process provides the security of our model so as to ensure that each user can access data safely.

**Figure 3:**
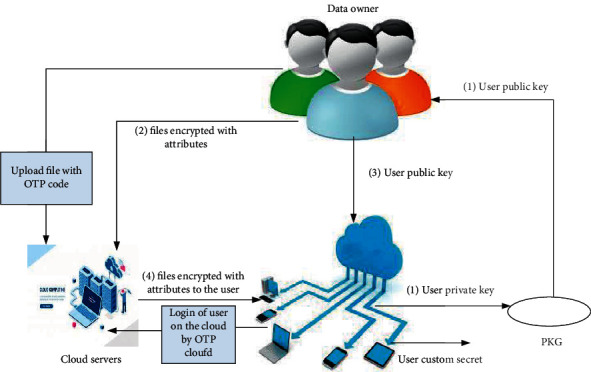
The proposed model.

The proposed model presents a solution to some security issues of cloud computing. Protection of authorized user from fake identity is one of the most important contributions to this model, because this issue has no solution before that, if it was necessary to try to solve this problem to maintain the trusted data access control for users of the system. Our proposed model provides security and scalability of data sharing for users on the cloud computing. All phases of the proposed model are combined in Figure 3.

## 4. Experimental Results

We implement our proposed model practically to achieve the security of cloud computing and provide good services to users of the cloud in any time and place. We execute this model using our simulation of the model that is NG-Cloud.

**Figure 4:**
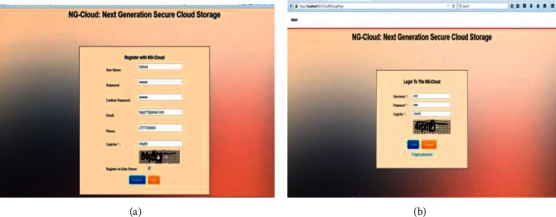
(a) Registration process. (b) Login process.

The NG-Cloud toolkit is a Java-based discrete-event cloud simulation toolkit that provides features for application composition, information services for resource discovery, and interfaces for assigning application tasks to resources and managing their execution. NG-Cloud website provides the maximum security protection techniques for end user and data owner. Such techniques are employed in login and registration process via CAPTCHA. Figures 4(a) and 4(b) illustrate the registration and login processes. In this step, each user or data owner can register in our system and type user name, password, e-mail, phone, and CAPTCHA. After the end of the registration process, every user or data owner can log into the cloud; when the data owner or the user log into the cloud, the cloud sends OTP code for them to enter this correct code to access the system.

**Figure 5:**
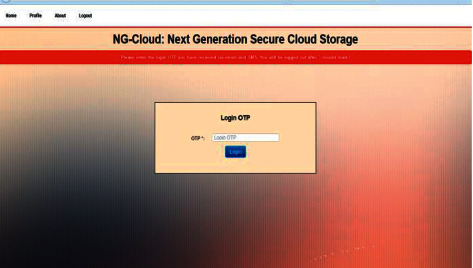
OTP code.

OTP code will be sent to the e-mail address and phone number to provide the authentication and security of the system. An example of OTP code to user or data owner is sent to their e-mail address as shown in Figure 5.

**Figure 6:**
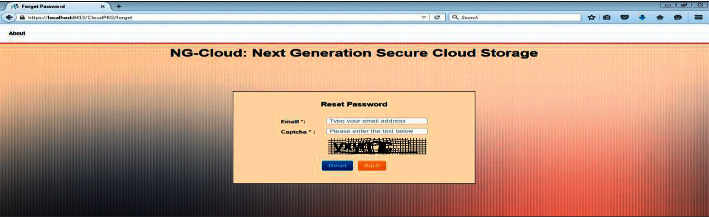
Rest password.

In Figure 6, for unauthorized access, authentication is required to be there in the cloud computing security structure. Thus, in case any user or data owner loses or by the data can be in danger, to protect the data, we have added the function Reset Password, which is a must to clear in order to access the data in cloud. Here, the user will restore her account, in which when user wants to restore his account, the user will type her e-mail and CAPATCHA text, and then the user can access her account on the cloud. So, the unauthorized user will face disappointment only even after having the correct user identity and password. Moreover, the attacker must know the master key to decrypt the encrypted data received from the cloud.

**Figure 7:**
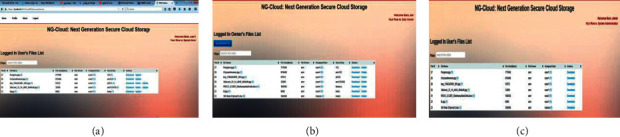
(a) User role, (b) data owner role, and (c) administrator role.

There are three roles in our implementation to manage the whole system to complete the main objective of the application, which is to provide excellent services to users over cloud computing. Figures 7(a)–7(c) illustrate the roles in NG-Cloud as follows: user, data owner, and administrator roles.

**Figure 8:**
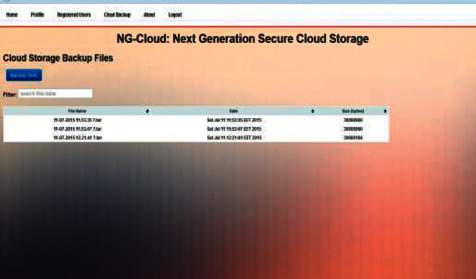
Cloud storage backup and Filter of keyword search.

In Figure 8, regarding cloud storage backup and filter of keyword search, deletion data without a backup, by loss of the encoding key, or by unauthorized access, data is always in danger of being lost or stolen. We add in our implementation function a cloud storage backup to protect the data from being lost, stolen, mistaken or leaked. Achieving keyword search is an extremely important function in our application, so we add the function filter of keyword search to search for matching words in the data.

**Figure 9:**
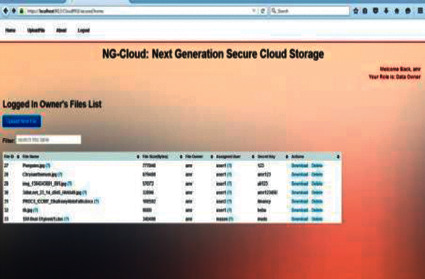
Privileges of data owner for users.

The data owner gives privileges for each user in order to use it in any time and in any place. The data owner can revoke any privileges when he wants. These privileges are Delete Download and Update as shown in Figure 9, privileges of data owner for users.

Moreover, exchanged data between owner and end user is encrypted using symmetric cryptography, and the key itself is encrypted using asymmetric cryptography. The uploaded data is secured between data owner and system user using common symmetric key, initiated at the beginning of the upload process.NG-Cloud provides strong encryption of the data by using symmetric encryption (AES algorithm) and asymmetric encryption (RSA algorithm). NG-Cloud solves the problems of fake identity and balancing balance security and usability. In this implementation, the data owner gives privileges to the user such as update, download, and delete and can revoke these privileges from users when he wants for any reason.

We implement NG-Cloud: next generation secure cloud storage on 100 users. 90 users are authorized, and 10 unauthorized. Our implementation: not allowed to enter any user and not allowed to enter the system and *d* detects these unauthorized users that are not allowed to enter the system. So, there is strong authentication of our system to protect users from any attacker on account of users. We will upload the files depending on the number of data owners, so that the system can be done smoothly and flexibility. For example, if we have two data owners who will be uploading files every 30 seconds or less and when it overcomes the 100 data owners, for example, each data owner will be downloading files every 5 minutes.

## 5. Analysis of the Proposed Model

An efficient cloud data security model should be able to overcome all the possible issues of cloud computing. We would like to provide the benefits of cloud computing without any troubles to propel in the direction it is designed for. This is to be achieved by preventing the owner's data from all the risks associated and provide a cloud model that is more secure and efficient. The proposed model shall overcome the security risks defined by the security functions over cloud computing as we listed [25] as follows:

1. Identification and authentication: the role of PKG is necessary to verify strong authentication. The issue authentication is very important in cloud computing environment [26]. PKG gives data owner user public key (user ID), whereas the data owner uploads his/her files to the assigned users, and therefore, each data owner should have user ID for each user. PKG grants every user in the system private key to decrypt the encrypted data. PKG protects the authenticated user, even if an external user had the user ID and password, from any attack, as the data requires the owner's private key to be decrypted. PKG is a trusted third party between the user and the data owner to avoid issues of authentication.
2. Authorization: authentication and authorization are common challenges in cloud computing [27]. Our model overcomes the problem of authorization by having the data owner set the user authorization privileges. The data owner will have tools to grant users privileges such as delete or update. Data owner can revoke these privileges from any user in the system any time. Data owner authorizes users with privileges to access the data. Without these privileges or permissions, users will be unauthorized on the cloud system. Data owner will control the process of authorization to prevent the authorization issues.
3. Confidentiality: in this model, we achieve the security function of confidentiality to protect the personal information of user. This is related to authorization, as confidentiality of data is maintained when only allowing authorized users from accessing the data on the cloud. Consequently, as users receive authorization privileges from data owners, the owner himself controls the confidentiality of their data. The only person who has the right to give or revoke user's specific privileges is the data owner. Data owner will put strict restrictions when users revoke their privileges, and those users cannot access the cloud again.
4. Nonrepudiation: we implement nonrepudiation to increase the security of the system. We protect the uploading/downloading phase of any user, so that he cannot deny any access to the system. Data owner uploads their encrypted file with a secret key on the cloud and then gives the user the secret key to decrypt encrypted file. Data owner assigns users for each file, and any access is logged and not denied.
5. Integrity: the issues of integrity are when unauthorized user changes the data. We solve the problem of integrity through data owner privileges, where data owner grants privileges to only authorize users. Authorized user shall have a private key through, in which he may decrypt the encrypted data, so the unauthorized user cannot alter the protected data.
6. Encryption: the main purpose of the data encryption process is to reduce the difficulty of understanding the content of the message with the difficulty of detecting the encryption mechanism [28]. Data owner encrypts data by secret key, and then cloud sends it to “User Secret Key” to user to access the encrypted data. Each user has public and private key by asymmetric key (RSA Algorithm); the public key and private key are different. In our model, any user cannot decrypt the file without the private key, so when the user wants to decrypt their data, he should have user secret key and private key; thus, an attacker will not be able to decrypt the data. We provide the protection of data by accurate encryption.
7. Storage provider verification: storage providers allow people and organizations to buy space to store their data. The storage provider has to be verified or is untrusted. This is considered the most important feature in any provider. The model will overcome the problem of storage provider verification by putting strict conditions to choose the provider to be a trustworthy person such as honesty, reliability, and fidelity and has a conscience springy. In case of ensuring that the provider is not a trusted person, we will be able to take the decision not to sell storage space to him.
8. Secure even after loss of user identity and password: we provide the security of our system for after loss of user identity or password to be secure. We provide the system by option of forget password to help the user access the system when he forgets or loses the user identity or password.
9. Indexing of data: data are acquired nowadays in huge amounts from different resources in a fast paste increasing the amount of data transferred within the cloud leads to system overloading and reduces the sufficiency of the system. Data transferred is not adequately shielded from different types of attacks that are encountered during transmission [29]. Data owner uploads data on the cloud and then assigns rights to users to download these data, and therefore, data owner can reduce the amount of data transferred through controlling the number of users allowed to download data. Indexing will facilitate the deployment of database back-end applications whether on private cloud or public cloud.
10. Keyword search: a type of search that looks for matching documents that contain one or more keywords specified by the user. Some researchers addressed this issue and tried to solve problems such as help users find their documents. We implemented the keyword search by adding options to the search query. The options help in filtering data that is presented in a table to facilitate the search process in short time. Data are stored in the cloud, and each user can search for matching documents that contain the given words. We will store the data by easy way, so that users can search for certain words in the documents.
11. Scalable data sharing: cloud systems can be used to enable data sharing capabilities, and this can provide abundant benefits to the user. There is currently a push for IT organizations to increase their data sharing efforts.
12. When the information is reencrypted, we have to distribute the brand new key to the closing customers with inside the organization and that is computationally inefficient and locates an excessive amount of burden at the statistics proprietor whilst thinking about massive organization sizes that might be in extra hundreds of thousands of customers. Hence, this answer is impractical to be deployed with the real-global for terribly critical data together with business, authorities, and/or clinical associated information.
13. There is now a growing focus on implementing scalable data sharing capabilities in the cloud. In the model, we shall provide Scalable Data sharing function, where many users can share data via the model, where every user has privileges via data owner, so many users can share the same data in the same time on the cloud.
14. Privacy: the issues of privacy are very important, because it pertains the sensitive data of users [30]. In this model, we shall provide privacy of users in the system and preserve the personal data from any breakthrough. Data owner stores the data in the cloud, and these data are encrypted by secret key. Each data owner will identify users with specific privileges for each uploaded file. Data owner sends user secret key of each file to user, and each user decrypts the file with user secret key and private key. Hence, every user will access only its own data, and so, we provide the function of privacy in the system.
15. Fake identity: security is a very critical issue in the cloud computing environment [11]. Authorized users may reveal their passwords to unauthorized users. Online computing is preferred by clients and businesses only if their data are assured to remain private and secure. This problem is very difficult, because the system cannot detect fake identity easily, where fake identity users will log into the system in a normal way. We shall solve this difficult problem by checking the real user's identity. Fake identity process is protected from bots and other automated programs by providing one-time password via mail and SMS jointly to the authorized user in the system. SMS is sent to the private phone number of user, and OTP is sent to the private mail of user to ensure the identity of authorized user.
16. Balance security and usability: increasing the usability of system provides risk over the system. We solve the issue of balancing security and usability through configuring the system. The process of configuration helps obtain balance between the security and usability. When we input Disable OTP in login process, the output is system Disable OTP (No OTP secrecy). When we input Disable OTP in file upload process, the output is system Disable OTP (No OTP secrecy). We will reduce the usability to maintain the security, as it is not inconsistent with the ability of the system in introducing best services for user.

Our implementation overcomes some potential threats on cloud computing. When we avoid these threats, any system will become more secure and safe. Data Loss/Leakage, Account, Service and Traffic Hijacking, Insecure Application Programming Interfaces, Abuse and Nefarious Use of Cloud computing, and Malicious Insiders are examples of threats of any system. When protecting the cloud environment from these threats and attacks, we will be able to provide the safety and security for users of cloud computing to enjoy all services and benefits of cloud computing.

## 6. Conclusion

Cloud computing has changed the way of delivering computing services. The proposed model presented a solution to some security issues of cloud computing. Protection of authorized user from any fake identity is one of the most important contributions to this model. In addition, the proposed model protects the system from any fake data owner who enters malicious data that may destroy the main goal of cloud computing services. Our proposed model provided the benefits and effectiveness of security in cloud computing. The proposed model solves the issue of balancing between security and usability. Also, the proposed model provided security and scalability of data sharing for users on the cloud computing. As future work, the proposed model shall be implemented over medical data records. New solutions of other threats and attacks can be found for its sensibility and importance. The performance and efficiency of the cloud-computing environment can be improved. It is required to propose a secure service composition to build a comprehensive policy-based management framework in cloud computing environments.

## Data Availability

The data used in the study can be obtained from the corresponding author upon your request.
